# Reply to Wouter K. Comment on “Lee et al. Influence of Aortoiliac Geometry on Non-Occlusive Thrombotic Risk Following Endovascular Repair of Abdominal Aortic Aneurysms. *Diagnostics* 2025, *15*, 2134”

**DOI:** 10.3390/diagnostics15202552

**Published:** 2025-10-10

**Authors:** Jeong In Lee, Dac Hong An Ngo, Hong Pil Hwang, Young Min Han, Hyo Sung Kwak

**Affiliations:** 1 Department of Medicine, Jeonbuk National University, Jeonju 54907, Republic of Korea; 2 Department of Radiology, Research Institute of Clinical Medicine of Jeonbuk National University—Biomedical Research Institute of Jeonbuk National University Hospital, Jeonbuk National University and Medical School, Jeonju 54907, Republic of Korea; ngodachongan@hueuni.edu.vn (D.H.A.N.);; 3 Department of Radiology, Hue University of Medicine and Pharmacy, Hue 530000, Vietnam; 4 Department of Surgery, Jeonbuk National University Hospital, Jeonju 54907, Republic of Korea

We would like to thank Dr. Wouter Kok for the thoughtful critique on our recently published article entitled: Influence of Aorto-Iliac Geometry on Non-Occlusive Thrombotic Risk Following EVAR.

We totally agree with you that the conclusion in our original article should emphasize on the fact that the relative value of vessel diameter or a difference in size between left and right iliac artery in each patient had higher importance in terms of thrombosis risk prediction. Our primary analyses compared thrombotic vs. non-thrombotic limbs and demonstrated associations between larger post-EVAR limb diameter, lower graft-limb and aorto-iliac angles, and greater iliac tortuosity with iliac thrombus. We also performed paired analyses within the 18 patients who developed unilateral thrombus, which again showed significant side-to-side differences in diameter, angles, and tortuosity [1]. In detail, the 18 thrombosed iliac arteries were larger than their contralateral arteries by 3.339 mm (*p* = 0.012) (Table 3 [1]). Another paired comparison in 36 patients with no thrombosis complication following EVAR showed no significant difference between both iliac artery (*p* = 0.12). Considering the above results, the relative size between two iliac artery post-EVAR maximal diameter was related to higher risk of thrombus formation. The grouping of all non-thrombus iliac artery without selectively compare with the contralateral side might create bias and inconsistency in our study. While post-EVAR limb diameter can be affected by multiple factors, including pre-intervention vessel size, device selection and oversizing and post-implant remodeling. Absolute post-EVAR diameter can be misleading on its own. A recent multicenter analysis linked oversizing (~19%) and iliac tortuosity to higher risk of iliac limb occlusion [2]. Other studies recommended against excessive (>~20%) oversizing, balancing it against the competing endoleak/migration risks [3,4]. Taken together, these data align with the fact that relative size (side-to-side), rather than absolute size alone, influence thrombosis risk.

Comparing to other studies in the same topic, our results were relatively different from those of Draper et al. [5]. We believe that a thorough comparison between pre- and post-EVAR geometry values would enlighten this controversial finding. For future analytic directions, we would like to thanks commenter for suggesting comparison between pre- and post-intervention vessel dimension. However, one of our study design limitation is that lack of pre-intervention data. We will consider this brilliant idea for our future research, a prospective, paired design reporting pre- vs. post-EVAR diameters, oversizing percentages, and geometric asymmetry indices across both thrombotic and non-thrombotic patients.

In summary, we appreciate the emphasis on paired comparison values. Our data already show that geometry (angles/tortuosity) and diameter are associated with thrombosis; we agree that relative size and oversizing likely modulate that risk and merit explicit reporting alongside absolute diameters.

## References

[1] Lee J.I., Ngo D.H.A., Hwang H.P., Han Y.M., Kwak H.S. (2025). Influence of Aortoiliac Geometry on Non-Occlusive Thrombotic Risk Following Endovascular Repair of Abdominal Aortic Aneurysms. Diagnostics.

[2] Yuan Z., Du C., You Y., Wang J. (2024). Predictive Factors for Iliac Limb Occlusions After Endovascular Abdominal Aneurysm Repair: Determined from Aortoiliac Anatomy, Endovascular Procedures, and Aneurysmal Remodeling. Ther. Clin. Risk Manag..

[3] Basra M., Hussain P., Li M., Kulkarni S., Stather P.W., Armon M., Choksy S. (2024). Factors Related to Limb Occlusion After Endovascular Abdominal Aortic Aneurysm Repair (EVAR). Ann. Vasc. Surg..

[4] Roos H., Sandström C., Koutouzi G., Jeppsson A., Falkenberg M. (2017). Predisposing Factors for Re-interventions with Additional Iliac Stent Grafts After Endovascular Aortic Repair. Eur. J. Vasc. Endovasc. Surg..

[5] Draper K., Choi S.H., Fung A., Baxter K., Taylor D., Chen J.C., Misskey J. (2023). Evaluation of factors associated with limb thrombus formation after endovascular aortic aneurysm repair. J. Vasc. Surg..

